# MAFLD: from a disease framework to patient care

**DOI:** 10.1007/s12072-024-10685-3

**Published:** 2024-06-17

**Authors:** Mohammed Eslam, Jacob George

**Affiliations:** Storr Liver Centre, The Westmead Institute for Medical Research, Westmead Hospital and University of Sydney, Westmead, NSW Australia

**Keywords:** MAFLD, MASLD, Metabolic dysfunction, Liver, Hepatology

**Fig. 1:**
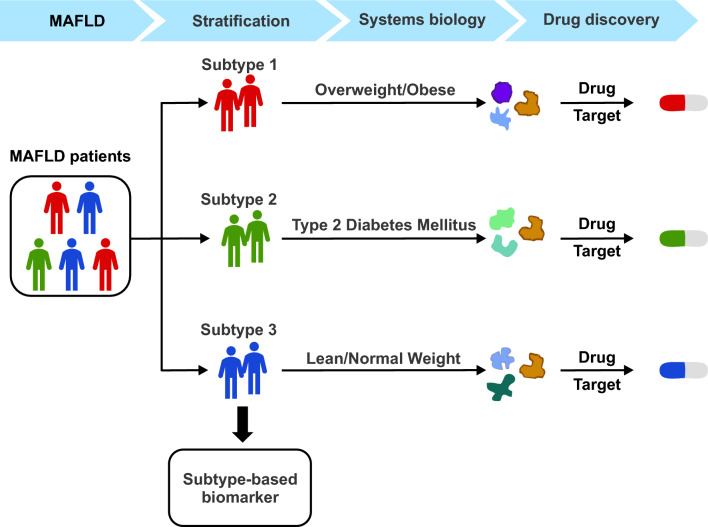
The utility of the MAFLD criteria for clinical practice

The year 2020 marked a watershed for Hepatology with an international panel suggesting a radical framework for the diagnosis of people with fatty liver disease related to metabolic dysfunction. The panel headed by experts from the Asia Pacific led to the introduction of the term metabolic dysfunction-associated fatty liver disease (MAFLD) and a novel diagnostic classification [1, 2]. The latter divided patients with MAFLD into three relatively homogenous groups—those who had steatosis and were overweight or obese according to ethnic-specific BMI criteria, those who had type 2 diabetes, and those who were of healthy weight but had two or more specified metabolic risk criteria (Figure 1). Subsequently, in the same year, APASL published the first global consensus guidelines on MAFLD [3], which was rapidly followed by guidelines from other national and pan-national societies and patient groups [4–7]. Since MAFLD can occur at any stage of the life span, a pediatric definition was introduced with age-appropriate criteria [8].

In the 4 years since publication, MAFLD has now been embedded in the literature with many thousand papers published on its clinical features, progression, and outcomes [9, 10]. So why has MAFLD been so invigorating for the field? First and foremost, MAFLD was introduced at the right time for a discipline that appreciated the anachronism of labelling a disease by what it was not, but had too much pent-up inertia to do anything about it. Refreshingly, MAFLD proposed that we diagnose the condition based solely on positive criteria. It was further emphasised that the presence of other liver diseases in the same patient should not detract from the simultaneous co-existence of MAFLD [11, 12]. This is critically important since we know that more than a third of the adult population have MAFLD, far in excess of the typically < 10% (and usually < 2%) prevalence of other liver diseases such as viral hepatitis or autoimmune liver disease [13, 14]. Hence, more commonly than not, MAFLD will co-exist with other liver diseases with bidirectional impacts on the clinical presentation, disease trajectory and outcomes [11, 12].

Alcohol-related liver disease is more common, but the amount of alcohol that begets liver disease has always been contentious [15]. Very high levels of alcohol consumption is an accepted cause of cirrhosis and liver cancer. But what about lower levels of consumption? The previous NAFLD definition (now relegated to history) suggested that alcohol consumption of 20 g/day in women and 30 g per day in men is acceptable, but that any level of alcohol consumption above these limits rendered a diagnosis of NAFLD invalid [15]. However, a wealth of literature suggests that levels even as low as 10 g/day results at least in hepatic steatosis and may contribute to disease progression and cancer [16]. If we accept the possibility that alcohol at low levels might impact the liver (or that the question is unresolved), does that not argue to omit any reference to alcohol in a definition of a liver disease that is due to systemic metabolic dysregulation? Conversely, if an individual has poorly controlled type 2 diabetes but consumes 40 g of alcohol a day, does that mean he/she does not have a component of their liver pathology related to metabolic dysregulation? Based on these relevant considerations in the clinical realm, the MAFLD framework is based solely on positive criteria—all individuals that meet the criteria have MAFLD, irrespective of any other concomitant liver disease [1, 15].

Some authors have argued for fatty liver disease to have an arbitrary threshold of alcohol as acceptable and to create a new entity called MetALD when this level is exceeded. Such a proposal detracts from diagnosing liver diseases based on positive attributes, but more importantly creates artificial and non-existent boundaries while hindering clinical research. The reality in practice is that any patient with MAFLD may consume alcohol at any level and this can vary over time. Both components—metabolic dysfunction and alcohol—across the continuum of each impacts the liver phenotype in various ways, including based on genetic predisposition. In other words, when an individual has metabolic dyfunction and alcohol consumption, there are two etiological factors (dual etiology liver disease) impacting the liver and we cannot “consensus” ourselves away from scientific truth.

The MAFLD proposal is, above all else, “clean” and reflects the reality of nosology. Clinical research can study the interaction between any level of metabolic dysregulation and any level of alcohol and determine the impact on the liver at a population level or at an individual patient level. The latter will become increasingly possible in the era of “omics” and artificial intelligence. To take an extreme example, if a patient with MAFLD consumes > 40 g of alcohol today, he/she has MetALD, and from tomorrow if he/she reduces consumption to 10 g per day he/she has MAFLD, a separate entity. In reality the patient has always had MAFLD. It has been argued that having “acceptable” limits of alcohol (20 or 30 g per day in men and women) aligns with prior epidemiological data and clinical trial entry criteria. Simply put, earlier epidemiological data indicates that fatty liver disease is highly prevalent and increasing, a conclusion that is not altered by a more appropriate conceptual framework and definition. A misrepresentation regarding alignment to trials criteria also exists [15, 17]. MAFLD trials set a limit for alcohol consumption and, for that matter, for BMI (most trials have considered individuals with BMI of 25 mg/kg^2^ or more) as part of their inclusion criteria. When a medication is approved for clinical use, it is for the clinician to decide if the patient has a disease related to metabolic dysfunction; if approval criteria mandates alcohol consumption below a particular limit or for that matter a particular BMI, then so be it. This does not detract from a MAFLD diagnosis, but rather only dictates the indications for use of an approved drug.

There is also an erroneous debate around stigma in the literature. While stigma is the first thing we should avoid, as recognised by others in the field and patient groups, the term “fatty liver” when used to describe a liver with fat is not stigmatising [18, 19]. More importantly, at the coalface, patients and indeed many health professionals do not understand the term “steatosis” a Latin term, which also flies in the face of the notion that we reduce medical jargon when conveying clinical messages to patients. Invariably, when a patient is told they have hepatic steatosis, the first question is “what does steatosis mean” to which our answer (and daresay of most clinicians) is that you have a “fatty liver”. For disease understanding and for patient ownership, messages should be conveyed simply and succintly [19].

So, what about the other attributes of MAFLD stemming from a positive diagnosis? In large parts of the world, the greatest scourge for liver disease and liver cancer has been viral hepatitis. This is particularly so in Asia, Africa and South America. MAFLD allows these diseases to be treated on their own merits, while also enabling a diagnosis of MAFLD, exactly as we do when a patient has both hepatitis B and C [20]. This also fits well with published data now indicating that the trajectory of those with viral hepatitis is different from those with viral hepatitis and MAFLD [9, 12]. While effective treatments are available for the former, the co-existence of MAFLD is a call to action for clinicians treating these patients. Not only must the liver disease (MAFLD) be managed, so also the competing risks of systemic metabolic dysregulation and its cardiorenal and extra-hepatic cancer consequences which undoubtedly kill more patients than their liver disease.

Secondly, a historical trend in human development has been increasing specialisation and so has it been in the field of medicine. We have gone from “heart failure” to heart failure with reduced ejection (HFrEF), heart failure with preserved ejection (HFpEF) and heart failure with mid-range ejection fraction (HFmrEF), each highlighting different clinical pathologies and approaches to treatment. So too, MAFLD has enabled the transition from a highly heterogeneous disease under the one rubric (NAFLD) to three sub-groups (overweight/obese, type diabetes, lean with metabolic dysfunction) with studies already demonstrating that each subgroup has different trajectories and outcomes in relation to all-cause, cardiovascular-related and cancer-related mortality, as well as in liver fibrosis stage and liver outcomes [21, 22]. In contrast, MASLD opts for the prevailing heterogeneity over homogeneity and the unique opportunity the latter enables for better defining disease course and, perhaps, treatment approaches. As an example, the obese phenotype might benefit most from a weight-centred pharmaceutical approach, while those who are of a healthy weight might benefit with more emphasis on improvements in fatty acid oxidation and reducing de novo lipogenesis, with less emphasis on weight loss.

What about the criteria used to define MAFLD? A unique difference is that in healthy weight individuals with MAFLD, there is a requirement for the presence of at least two metabolic risk criteria, including measures of insulin resistances and cardiometabolic inflammation. In contrast, MASLD—a liver disease—only requires the presence of a single cardiometabolic risk factor for diagnosis and, for example, there is no evidence that hypertension or a low HDL by itself results in long-term adverse liver-related outcomes. Since the prevalence of at least one cardiometabolic risk factor is high (> 90%) in older adults and is equally high (85%) in those without steatosis, there is low specificity of this diagnostic criterion. From a pathophysiological perspective, insulin resistance, as we described [23], is a cardinal manifestation of MAFLD, while for low HDL and diastolic BP, the link to insulin resistance and steatosis is weak. Finally, the MAFLD criteria has a clear definition for what constitutes MAFLD cirrhosis enabling future clinical research.

Through the MAFLD journey, a lot of water has flowed under the bridge; much has been learnt, while a lot more needs to be discovered. This is particularly with regard to the etiopathogenesis of liver cancer, especially in those who do not have cirrhosis, and on the mechanistic underpinnings of MAFLD in individuals of a healthy weight. We also do not fully understand the determinants of differential disease trajectories of the various sub-groups of MAFLD. While it is impossible to cover all there is to MAFLD, the Editors of this supplement have chosen a selection of 12 topics that first and foremost will appeal to the practising clinician. These cover the natural history of MAFLD across the life span and its association with extra-hepatic outcomes, since the liver is just one organ targeted by a systemic process that affects all organs and tissues in different ways. We cover the unique situations when MAFLD comes into its own—coalescing homogenous patient sub-groups (MAFLD in healthy weight individuals as an example), and those with the major subtypes of dual etiology liver disease. From a pathophysiological perspective, we have sought to restrict coverage to the role of microbiota, genetic and epigenetic factors, and immune cells. Regarding management, we cover disease staging and monitoring, which will become increasingly important as the scale of the epidemic is realised and the hepatology workforce is overwhelmed. New tools for disease staging (either liquid biopsy or imaging based) will become critical to ensure that those most in need of our expertise get the care they deserve without the need for invasive liver biopsy. We devote two chapters, one each to lifestyle-centred MAFLD management and one to the pharmacotherapies that will soon surely be approved. Finally, liver cancer and acute on chronic liver failure, among the most feared complications of MAFLD that heralds the increasing use of hospital resources, societal and family burden, will be addressed. We hope this will be the start of many future supplements on MAFLD, a concept and a diagnostic framework that has changed how we practice Hepatology the world over.
